# Elevated D-dimer is independently associated with in-hospital mortality in patients with *Klebsiella pneumoniae* bloodstream infection: a cohort study

**DOI:** 10.3389/fcimb.2026.1778926

**Published:** 2026-07-06

**Authors:** Xiao Dong, Ting Ao, Yingxiu Huang, Ming Hu, Peng Zhen

**Affiliations:** Department of Infectious Disease, Beijing Luhe Hospital, Capital Medical University, Beijing, China

**Keywords:** bloodstream infection, d-dimer, *Klebsiella pneumoniae*, mortality, prognosis

## Abstract

**Objective:**

Although D-dimer is a routine hematological parameter and has been associated with mortality in various diseases, its specific relationship with clinical outcomes in *Klebsiella pneumoniae* bloodstream infection (KP-BSI) remains insufficiently explored. This study aimed to investigate the association between D-dimer levels and in-hospital mortality in patients with KP-BSI.

**Methods:**

We conducted a retrospective cohort study of adult patients with KP-BSI admitted to a tertiary hospital between 2019 and 2024. The primary outcome was in-hospital mortality. Multivariable Cox regression was employed to assess the independent association between D-dimer levels and mortality risk, with additional subgroup and sensitivity analyses performed to verify the robustness of the findings.

**Results:**

A total of 222 patients (156 male, 70.3%; mean age 66.4 ± 13.6 years) were enrolled. Among them, 106 (47.7%) died during hospitalization. After full adjustment for potential confounders, elevated D-dimer levels were independently associated with an increased risk of in-hospital mortality (adjusted hazard ratio [HR] 1.04, 95% confidence interval [CI] 1.03–1.07, P = 0.001). When analyzed categorically using a 5 mg/L, patients in the high D-dimer group showed a significantly higher mortality rate compared to those in the low D-dimer group (HR: 2.14, 95% CI: 1.27–3.59; P = 0.004). The results remained consistent across subgroup and sensitivity analyses.

**Conclusion:**

High D-dimer levels are independently associated with increased in-hospital mortality in patients with KP-BSI, suggesting its potential utility as a prognostic biomarker in this population.

## Introduction

*Klebsiella pneumoniae* bloodstream infection (KP-BSI) represents a major global health threat, characterized by substantial morbidity and mortality. In 2019 alone, infections attributable to *K. pneumoniae* were associated with an estimated 790,000 deaths worldwide (Hwang et al., 2025). Patients who develop KP-BSI are often hospitalized with significant comorbidities (Abbas et al., 2024), facing an in-hospital mortality rate as high as 14.4% (Roach et al., 2024). The clinical challenge is further compounded by rising antimicrobial resistance. Surveillance data from Europe indicate that 12 out of 41 countries reported carbapenem resistance rates exceeding 25% among *K. pneumoniae* isolates, with rates reaching 50% or higher in six nations (Wang et al., 2019). This alarming landscape underscores the critical need for reliable tools to identify high-risk patients early, enabling timely intervention and optimized care.

Current strategies for prognostic assessment in sepsis rely heavily on clinical scoring systems like Sequential Organ Failure Assessment (SOFA) and conventional biomarkers of inflammation, including procalcitonin and C-reactive protein(CRP) (Gao et al., 2022; Hertz et al., 2022). While useful, these tools possess inherent limitations (Zheng et al., 2020). Their specificity can be suboptimal, their dynamic range may not fully capture rapid clinical changes, and importantly, they do not directly reflect the profound coagulation dysfunction that is central to sepsis pathophysiology. Therefore, identifying novel, readily available biomarkers that integrate broader aspects of disease severity, including the hematological axis, remains a priority.

D-dimer is a specific terminal degradation product of cross-linked fibrin. It is therefore recognized as a direct biochemical indicator of both coagulation activation and subsequent fibrinolysis (Zhang et al., 2015; Han et al., 2021). In severe infection, the systemic inflammatory response triggered by pathogens can induce endothelial damage, leading to disproportionate activation of the coagulation cascade and impaired fibrinolysis—a process termed immunothrombosis. This state contributes significantly to microvascular thrombosis and subsequent organ dysfunction (He et al., 2022). Elevated D-dimer levels have been consistently and independently linked to worse outcomes, including mortality, in diverse infectious conditions like sepsis (Han et al., 2021), community-acquired pneumonia, and COVID-19 (Ghanem et al., 2023). However, its specific role as an independent prognostic predictor in the distinct context of KP-BSI has not been clearly established.

This study aimed to clarify this issue by retrospectively evaluating whether D-dimer levels are associated with in-hospital mortality in a cohort of patients with KP-BSI. We hypothesized that an elevated D-dimer level at presentation would serve as an independent predictor of increased mortality in this patient population. Elucidating this relationship could provide a valuable, straightforward parameter for early risk stratification, potentially informing more personalized management strategies for patients with KP-BSI.

## Materials and methods

### Data source

This single-center, retrospective cohort study was conducted at Beijing Luhe Hospital, Capital Medical University, a tertiary care facility with 1,300 inpatient beds located in eastern Beijing, China. We systematically screened consecutive adult patients diagnosed with KP-BSI at the hospital between January 2019 and December 2024. The study protocol followed the principles of the Declaration of Helsinki and was approved by the Institutional Ethics Committee of Beijing Luhe Hospital, Capital Medical University (Approval No. 2025-LHKY-017-01). Due to the anonymous and retrospective nature of the data analysis, the requirement for informed consent was waived. The study was performed in compliance with applicable local regulations and institutional policies. This study was conducted and reported in accordance with the Strengthening the Reporting of Observational Studies in Epidemiology (STROBE) guidelines.

### Study population

**Figure 1:**
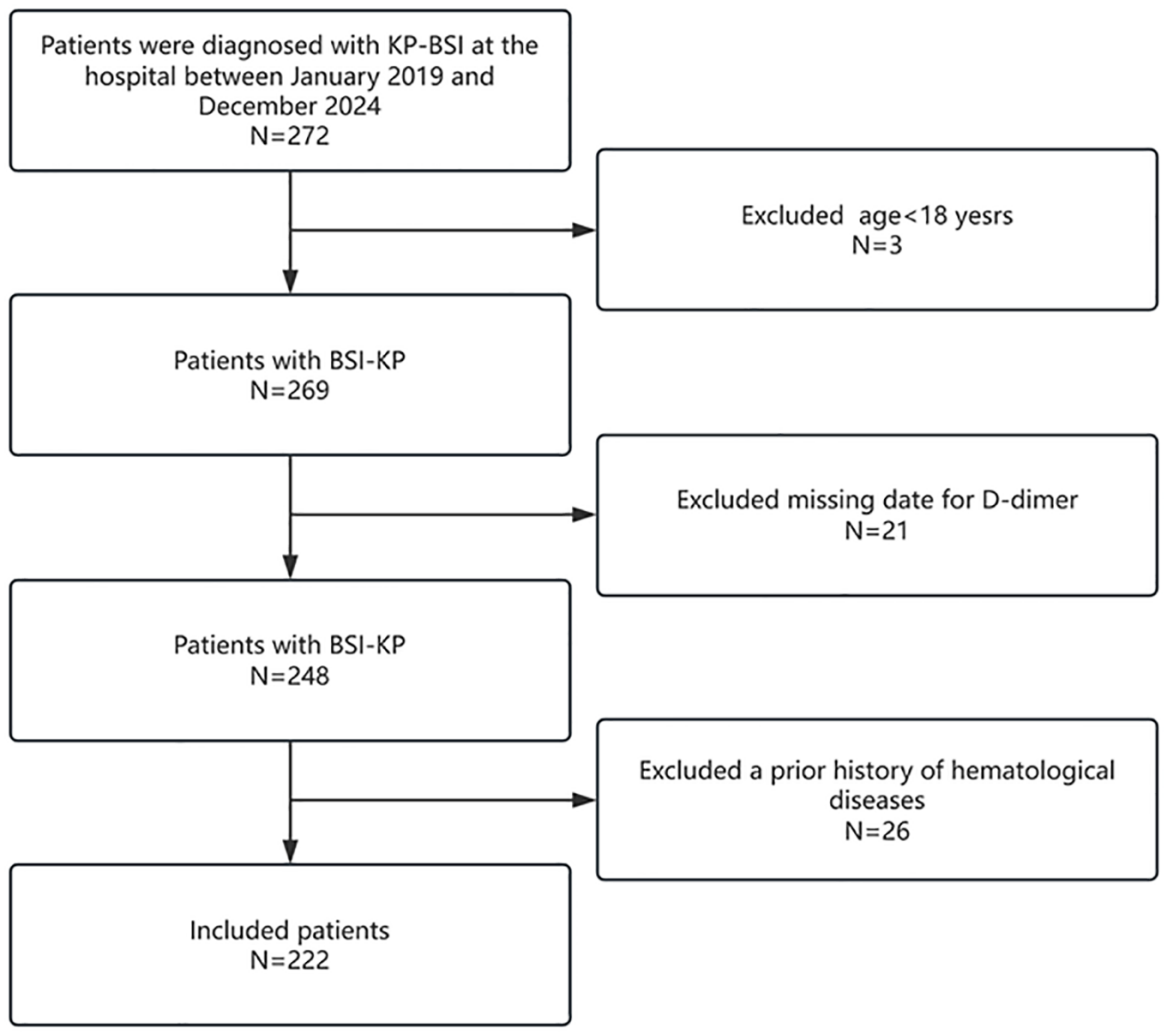
Flow chart of study. KP-BSI *Klebsiella pneumoniae* bloodstream infections.

Clinical data were extracted from the hospital’s electronic medical record system. All personally identifiable information was removed prior to analysis to ensure patient privacy. The inclusion criteria were as follows: (1) confirmed diagnosis of KP-BSI, defined as at least one blood culture positive for *K. pneumoniae*, accompanied by compatible clinical manifestations of bloodstream infection, with cases deemed to represent contamination excluded; (2) age over 18 years; (3) availability of complete blood count data. For patients with multiple episodes of infection, only the first episode was included in the analysis. Exclusion criteria included age under 18 years, incomplete clinical data, multiple KP-positive episodes (only the first episode was included), indeterminate infection diagnosis, and a prior history of hematological diseases. The patient screening and selection process is summarized in **Figure 1**.

### Microbiological tests

Species identification and antimicrobial susceptibility testing for all clinical isolates were performed using the VITEK 2 Compact automated system (bioMérieux, France). Susceptibility results were interpreted according to the current Clinical and Laboratory Standards Institute guidelines (CLSI M100) (Chen et al., 2024).

### Variable extraction

#### D-dimer

D-dimer levels were assessed within 24 hours following blood culture collection and recorded in the clinical database. For analysis, D-dimer was treated as a continuous and categorical variable. Based on a cut-off value of 5 mg/L for D-dimer (Semeraro et al., 2019; Han et al., 2021), patients were stratified into a low D-dimer group (<5 mg/L) and a high D-dimer group (≥5 mg/L).

#### Covariates

Demographic data (sex and age) and comorbidities (including diabetes, hypertension, chronic respiratory disease, cerebrovascular disease, chronic heart failure, chronic liver disease, chronic renal insufficiency, and malignancy) were extracted. Laboratory parameters—comprising white blood cell count (WBC), platelet count (PLT), CRP, procalcitonin, creatinine (Cr), blood urea nitrogen (BUN), albumin (ALB), total bilirubin, fibrinogen, activated partial thromboplastin time (APTT), lactate, base excess (BE), and oxygenation index—were measured and recorded within 24 hours after blood culture. Microbiological factors included the presence of carbapenem-resistant *Klebsiella pneumoniae* (CRKP). Disease severity was assessed using the SOFA score. Additional covariates comprised intensive care unit admission and the administration of appropriate antibiotic therapy during the initial phase of infection. Covariate selection for the multivariable models was based on clinical relevance and prior knowledge of confounding pathways in sepsis. Variables considered potential mediators of the effect of D-dimer on mortality (e.g., lactate, base excess) were deliberately excluded from the adjusted models to avoid overadjustment bias. The final model (Model 4) included variables hypothesized to confound the relationship between coagulation activation and mortality: demographic factors (age, sex), baseline comorbidities, markers of acute organ dysfunction (SOFA score), and management factors (inappropriate antibiotic therapy, CRKP status).

### Outcomes

The primary endpoint was all-cause mortality during the hospital stay.

### Statistical analysis

Descriptive statistics were calculated for all patients. Categorical variables are presented as frequency (percentage). Normality of continuous variables was assessed using the Shapiro-Wilk test and visual inspection of Q-Q plots. Continuous variables following a normal distribution are expressed as mean ± standard deviation (SD), while non-normally distributed variables are presented as median [interquartile range, IQR]. Comparisons between groups were performed using the Chi-square test for categorical variables, one-way ANOVA for normally distributed continuous variables, and the Kruskal-Wallis H test for non-normally distributed continuous variables. Covariates had less than 1% missing values; therefore, no imputation was performed.

The independent association between D-dimer and in-hospital mortality was assessed using Cox proportional hazards regression, with sequential adjustment across four nested models. In accordance with STROBE guidelines, Model 1 was unadjusted. Model 2 was adjusted for sex and age. Model 3 included further adjustments for laboratory parameters (WBC, CRP, ALB, APTT) and comorbidities (diabetes, malignancy, chronic liver disease, chronic renal insufficiency). Model 4 additionally adjusted for CRKP, SOFA score, admission to the intensive care unit, and inappropriate initial antibiotic therapy.

Kaplan-Meier survival curves were plotted, and the Log-rank test was used to compare survival differences across D-dimer quartiles. Subgroup and sensitivity analyses were conducted to examine the association within specific populations and to assess the robustness of the findings. Model 4 covariates were used consistently in all adjusted subgroup, sensitivity, and Kaplan-Meier analyses.

All statistical analyses were conducted using R software (version 4.3.2; R Foundation for Statistical Computing) and Free Statistics software (version 2.2). Statistical significance was defined as a two-sided P value of less than 0.05. No *a priori* sample size calculation was performed, as this was a retrospective study including all eligible patients over the study period. With 106 events, the study had >80% power to detect a hazard ratio of ≥2.0 for the primary categorical comparison at α=0.05.

## Results

### Baseline characteristics of participants

Our final analysis comprised a cohort of 222 patients with KP-BSI (**Table 1**). Among them, 106 patients (47.7%) died during hospitalization, while 116 patients (52.3%) survived. Compared with survivors, non-survivors exhibited more severe systemic infection and organ dysfunction. Regarding infection and inflammatory biomarkers, non-survivors had significantly higher levels of procalcitonin (median: 17.8 vs. 4.9 ng/mL, P < 0.001), CRP (184.0 ± 81.0 vs. 147.6 ± 85.1 mg/L, P = 0.001), and lactate (median: 5.1 vs. 2.1 mmol/L, P < 0.001). Notably, the median plasma D-dimer level was also significantly higher in non-survivors (2.5 vs. 1.1 mg/L, P < 0.001). In terms of organ function and metabolic parameters, non-survivors demonstrated worse renal function (creatinine: 156.0 vs. 84.5 μmol/L; BUN: 18.3 vs. 7.4 mmol/L, both P < 0.001), more severe metabolic acidosis (BE: -8.1 vs. -3.1 mmol/L, P < 0.001), and more pronounced hypoalbuminemia (albumin: 27.5 ± 17.9 vs. 31.7 ± 5.4 g/L, P = 0.017). Coagulation profiles were also more deranged in non-survivors, evidenced by lower fibrinogen levels (4.2 ± 2.0 vs. 5.0 ± 1.8 g/L, P = 0.001) and a longer APTT (44.1 ± 22.8 vs. 34.5 ± 10.7 seconds, P < 0.001). Furthermore, disease severity was significantly greater in non-survivors, as indicated by a substantially higher median SOFA score (10.0 vs. 3.0, P < 0.001). Regarding treatment-related factors, the proportions of patients with CRKP infection (44.3% vs. 19.0%, P < 0.001), admission to the intensive care unit (86.8% vs. 42.2%, P < 0.001), and inappropriate initial antibiotic therapy (50.9% vs. 24.1%, P < 0.001) were all significantly higher in the non-survivor group.

**Table 1:** Baseline characteristics and clinical outcomes of patients with *Klebsiella pneumoniae* bloodstream infection, stratified by survival status.

Variables	Total (n = 222)	Survival (n = 116)	Non-survival (n = 106)	p
Demographics				
Sex,Male, n (%)	156 (70.3)	82 (70.7)	74 (69.8)	0.886
AGE, Mean ± SD	66.4 ± 13.6	67.1 ± 13.6	65.6 ± 13.7	0.416
Laboratory parameters				
D-dimer, mg/L	1.4 (0.8, 3.5)	1.1 (0.6, 2.5)	2.5 (1.0, 4.8)	< 0.001
WBC, ×10^9^/L	10.3 (6.4, 15.9)	9.6 (6.6, 14.1)	11.8 (6.3, 19.4)	0.163
Platelet,×10^9^/L	145.2 ± 96.5	155.8 ± 89.7	133.7 ± 102.7	0.089
CRP, mg/L	164.9 ± 84.9	147.6 ± 85.1	184.0 ± 81.0	0.001
Procalcitonin, ng/mL	10.2 (3.2, 26.5)	4.9 (1.3, 15.6)	17.8 (6.7, 36.6)	< 0.001
Albumin, g/L	29.7 ± 13.1	31.7 ± 5.4	27.5 ± 17.9	0.017
Creatinine, umol/L	98.5 (64.2, 195.8)	84.5 (60.0, 142.2)	156.0 (84.2, 265.8)	< 0.001
BUN, mmol/L	11.6 (6.7, 20.8)	7.4 (5.2, 13.4)	18.3 (10.0, 27.9)	< 0.001
Total bilirubin, μmol/L,	16.1 (9.4, 45.0)	15.9 (9.5, 32.2)	16.4 (9.4, 61.8)	0.38
Fibrinogen, g/L	4.6 ± 2.0	5.0 ± 1.8	4.2 ± 2.0	0.001
APTT, s	39.1 ± 18.2	34.5 ± 10.7	44.1 ± 22.8	< 0.001
Lactate, mmol/L	3.6 (1.8, 6.0)	2.1 (1.4, 3.5)	5.1 (3.5, 8.1)	< 0.001
BE, mmol/L	-6.2 (-10.2, -1.2)	-3.1 (-6.5, 0.3)	-8.1 (-12.1, -4.4)	< 0.001
Disease severity				
SOFA score,	6.0 (2.0, 10.0)	3.0 (1.0, 5.0)	10.0 (8.0, 12.0)	< 0.001
Comorbidities, n (%)				
Diabetes	102 (45.9)	57 (49.1)	45 (42.5)	0.318
Hypertension	125 (56.3)	62 (53.4)	63 (59.4)	0.369
Chronic respiratory disease	25 (11.3)	10 (8.6)	15 (14.2)	0.193
Cerebrovascular disease	94 (42.3)	49 (42.2)	45 (42.5)	0.975
Chronic heart failure	33 (14.9)	13 (11.2)	20 (18.9)	0.109
Chronic liver disease	17 (7.7)	8 (6.9)	9 (8.5)	0.656
Chronic kidney disease	28 (12.6)	12 (10.3)	16 (15.1)	0.287
Malignancy	38 (17.1)	22 (19)	16 (15.1)	0.444
Clinical and microbiological features, n (%)				
CRKP	69 (31.1)	22 (19)	47 (44.3)	< 0.001
Inappropriate initial antibiotic therapy	82 (36.9)	28 (24.1)	54 (50.9)	< 0.001
Admission to intensive care unit	141 (63.5)	49 (42.2)	92 (86.8)	< 0.001

Categorical variables are presented as counts and percentages (n, %), normally distributed continuous variables as mean ± standard deviation (SD), and nonnormally distributed continuous variables as median with interquartile range (IQR). CRKP, carbapenem-resistant *Klebsiella pneumoniae*; WBC, white blood cell; CRP, C,reactive protein; BUN blood urea nitrogen; APTT, Activated partial thromboplastin time; BE, Base excess; SOFA, sequential organ failure assessment.

Categorical variables were compared using the Chi-square test. Normally distributed continuous variables were compared using one-way ANOVA and are expressed as mean ± SD. Non-normally distributed continuous variables were compared using the Kruskal-Wallis H test and are expressed as median (IQR).

### Association between D-dimer and in-hospital mortality

**Figure 2:**
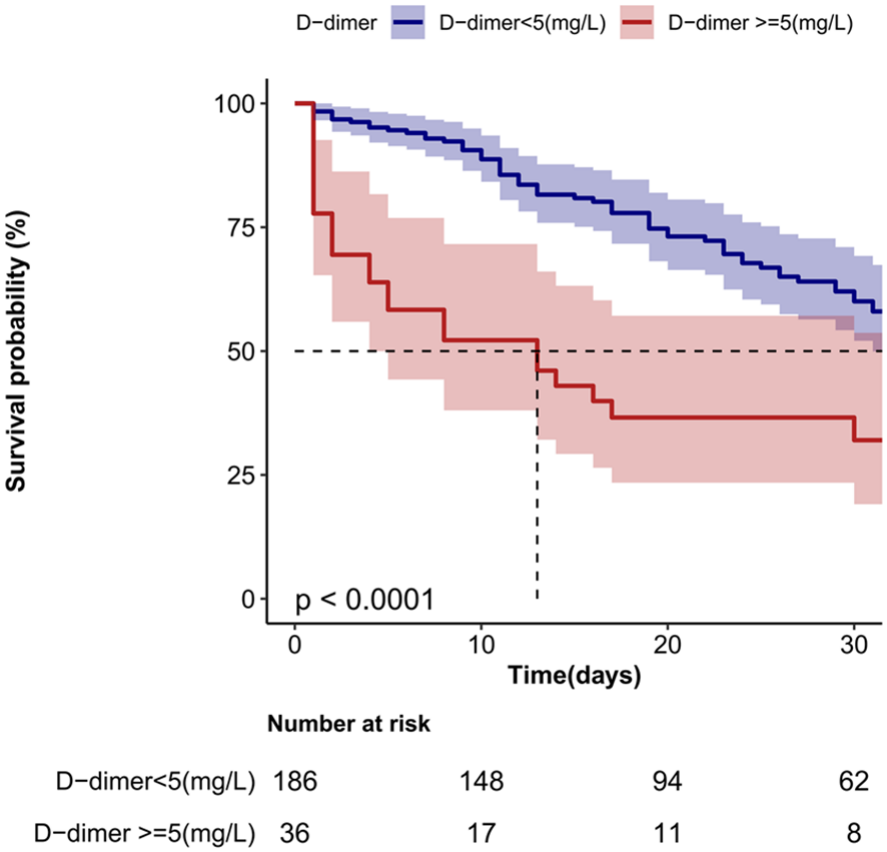
Kaplan-Meier Survival Analysis Stratified by D-Dimer Level. Kaplan-Meier curves showing cumulative survival in patients with KP-BSI, grouped according to D-dimer level at presentation (cutoff: 5 mg/L). Patients with D-dimer ≥5 mg/L had significantly worse survival compared to those with D-dimer <5 mg/L (Log-rank test, p < 0.0001). The number of patients at risk at each time point (0, 10, 20, 30 days) is shown in the corresponding table below the figure. KP-BSI*, Klebsiella pneumoniae* bloodstream infection.

A series of sequential models were constructed to assess the independent effect of D-dimer on in-hospital mortality in *KP-BSI* patients after adjusting for potential confounders (**Table 2**). Across the initial models, each 1 mg/L rise in D-dimer level conferred a heightened risk of mortality, evidenced by a consistent hazard ratio (HR) of 1.07 (95% CI: 1.05–1.09, P < 0.001) in both Model 1 (unadjusted), Model 2 (adjusted for sex and age) and Model 3 (further adjusted for laboratory parameters). In the fully adjusted model (Model 4), D-dimer remained an independent predictor, with each 1 mg/L increment corresponding to a 4% increase in the risk of in-hospital death (adjusted HR 1.04, 95% CI 1.03–1.07, P = 0.001). A categorical analysis revealed that mortality was significantly higher in the high D-dimer group relative to the low D-dimer group. In the unadjusted crude model, the HR for mortality in the high D-dimer group was 2.58 (95% CI: 1.64–4.06; P < 0.001). This association remained statistically significant in the fully adjusted Model 4 (HR: 2.14, 95% CI: 1.27–3.59; P = 0.004). Kaplan-Meier survival analysis (**Figure 2**) further confirmed a dose-dependent increase in cumulative mortality associated with elevated D-dimer levels (Log-rank P < 0.001).

**Table 2:** Risk of mortality in patients with *Klebsiella pneumoniae* bloodstream infection according to D-Dimer.

Variable	Model 1		Model 2		Model 3		Model 4	
	HR (95% CI)	p	HR (95% CI)	p	HR (95% CI)	p	HR (95% CI)	p
D-Dimer(mg/L) continues	1.07(1.05–1.09)	<0.001	1.07 (1.05–1.09)	<0.001	1.07 (1.05–1.09)	<0.001	1.04 (1.02–1.07)	0.001
Based on a cut-off value of 5 mg/L for D-dimer								
D-dimer <5(mg/L)	1(Ref)		1(Ref)		1(Ref)		1(Ref)	
D-dimer ≥5(mg/L)	2.58 (1.64–4.06)	<0.001	2.56 (1.63–4.03)	<0.001	2.33 (1.4–3.89)	0.001	2.14 (1.27–3.59)	0.004

HR, hazard ratio; CI, confidence interval.

Model Adjustments:.

Model 1: Unadjusted.

Model 2: Adjusted for age, sex.

Model 3: Further adjusted for laboratory parameters (WBC, CRP, Alb, APTT) and comorbidities (diabetes, malignancy, Chronic liver disease, Chronic renal disease).

Model 4: Fully adjusted (SOFA, Admitted to ICU, Inappropriate initial antibiotic therapy and carbapenem-resistant *Klebsiella pneumoniae*).

Statistical Conventions: HRs and 95% CIs rounded to two decimal places; p-values reported as “<0.001” if below 0.001.

HRs and 95% CIs were derived from Cox proportional hazards regression.

### Subgroup analysis

**Figure 3:**
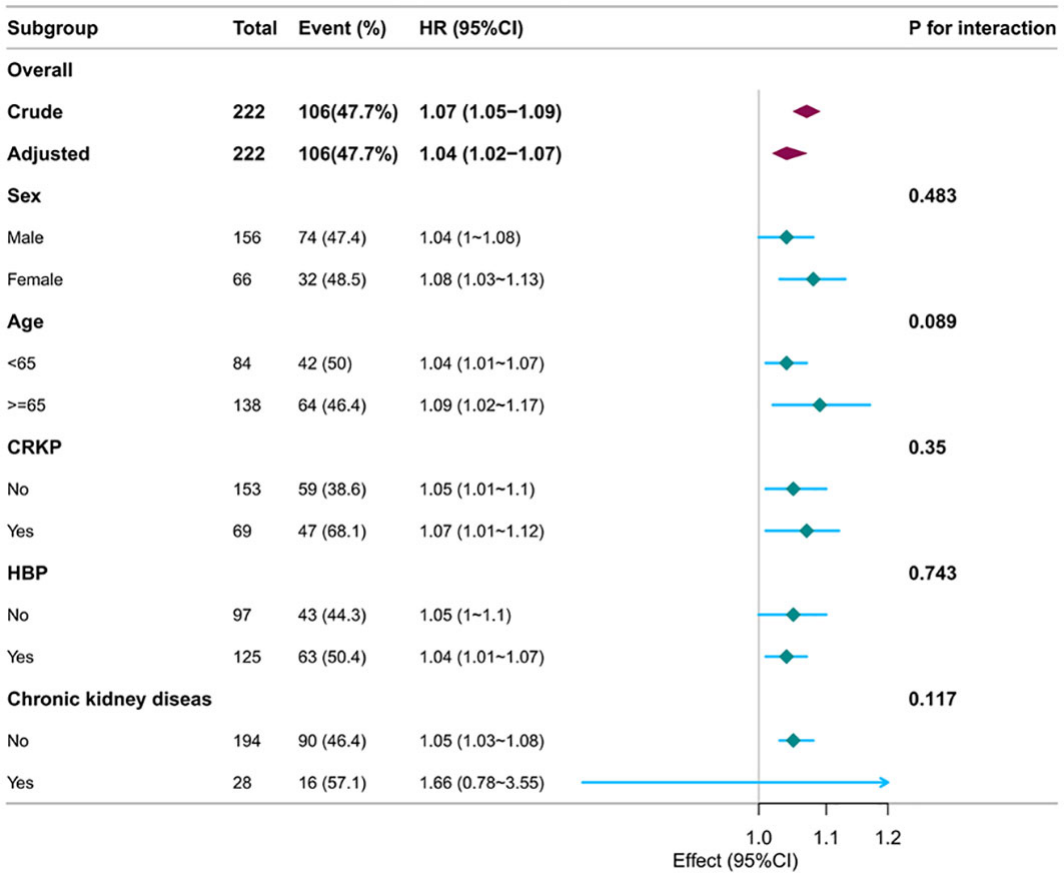
Forest plot of subgroup analyses for the association between D-dimer and in-hospital mortality in KP-BSI patients. Hazard ratio (HRs) were adjusted for age, sex, WBC, CRP, Alb, APTT, diabetes, malignancy, Chronic liver disease, Chronic renal disease, Admitted to ICU, Inappropriate initial antibiotic therapy and CRKP. KP-BSI, *Klebsiella pneumoniae* bloodstream infections; WBC, white blood cell; CRP, C-reactive protein; Alb albumin; APTT, activated partial thromboplastin time; CRKP, carbapenem-resistant *Klebsiella pneumoniae*.

Prespecified subgroup analyses were performed based on age (<65 or ≥65 years), sex, infection with CRKP, and the presence of hypertension or chronic kidney disease (**Figure 3**). Across all subgroups, elevated D-dimer levels remained significantly associated with increased in-hospital mortality (P < 0.05 for all comparisons). However, no statistically significant interaction was detected for any of the subgroup variables (P for interaction > 0.05 for all).

### Sensitivity analysis

A sensitivity analysis was performed to test the robustness of our results, Specifically, we repeated the primary analysis after the exclusion of patients diagnosed with malignant tumors or chronic liver disease. The significant association of D-dimer with in-hospital mortality was preserved in the sensitivity analysis, as detailed in **Table 3**. A *post-hoc* ROC analysis yielded an AUC of 0.666 (95% CI: 0.595–0.737; **Supplementary Figure 1**), with a Youden-derived optimal cutoff of 0.46 mg/L. Using this cutoff, D-dimer ≥0.46mg/L was associated with mortality in univariate analysis (HR 3.02, 95% CI 1.22–7.45, P = 0.017) but not after multivariable adjustment (adjusted HR 0.76, 95% CI 0.27–2.11, P = 0.596; **Supplementary Table 1**).

**Table 3:** Sensitivity Analysis.

Variable	N.total	n.event_%	Crude model		Adjusted model	
			HR (95% CI)	p	HR (95% CI)	p
Excluding patients with malignancy						
D-dimer(mg/L) continues	184	90 (48.9)	1.07 (1.05~1.09)	<0.001**	1.05 (1.02~1.07)	0.001
Based on a cut-off value of 5 mg/L for D-dimer						
D-dimer <5(mg/L)	152.0	68 (44.7)	1(Ref)		1(Ref)	
D-dimer ≥5(mg/L)	32.0	22 (68.8)	2.49 (1.53~4.04)	<0.001	2 (1.15~3.48)	0.014
Excluding patients with chronic liver disease						
D-Dimer(mg/L)	205	97 (47.3)	1.07 (1.05~1.09)	<0.001	1.04 (1.01~1.06)	0.002
Based on a cut-off value of 5 mg/L for D-dimer						
D-dimer <5(mg/L)	173.0	76 (43.9)	1(Ref)		1(Ref)	
D-dimer ≥5(mg/L)	32.0	21 (65.6)	2.43 (1.49~3.95)	<0.001	2.13 (1.23~3.69)	0.007

HR, hazard ratio; CI, confidence interval.

Adjusted Model: Adjusted for age, sex, laboratory parameters (WBC, CRP, Alb, APTT), comorbidities (diabetes, malignancy, Chronic liver disease, Chronic renal disease), Admitted to ICU, Inappropriate initial antibiotic therapy and carbapenem-resistant *Klebsiella pneumoniae*).

Statistical Conventions: HRs and 95% CIs rounded to two decimal places; p-values reported as “<0.001” if below 0.001. HR, hazard ratio; CI, confidence interval.

HRs and 95% CIs were derived from Cox proportional hazards regression.

## Discussion

This study provides the first comprehensive evidence of an independent positive association between D-dimer levels and in-hospital mortality in patients with KP-BSI. Our results demonstrate that after multivariable adjustment, each 1 mg/L increase in D-dimer was associated with a 4% increase in the risk of in-hospital death (HR 1.04, 95% CI 1.03–1.07, P = 0.001). This association was consistent across various predefined clinical subgroups. Moreover, the relationship remained robust in sensitivity analyses that excluded patients with specific comorbidities known to affect coagulation, such as malignancy or chronic liver disease. Collectively, these findings indicate that plasma D-dimer level is an independent predictor of mortality in patients with KP-BSI.

D-dimer is a degradation product of cross-linked fibrin. Fibrinogen is cleaved by thrombin to form fibrin monomers, which spontaneously polymerize in a half-staggered, overlapping manner to produce unstable, soluble fibrin polymers. These soluble polymers are subsequently covalently cross-linked by activated factor XIII (FXIIIa) to form insoluble, cross-linked fibrin polymers (Johnson et al., 2019; Chinese Society on Thrombosis and Hemostasis, 2023). Cross-linked fibrin polymers are degraded by plasmin, yielding substantial amounts of complex, long-chain X-oligomers. These large fragments are further cleaved into a mixture of variously sized fragments containing two covalently linked D domains (D=D, representing the D-dimer molecule), with the final terminal product being D=D/E. Since D-dimer is generated exclusively during the degradation of cross-linked fibrin, it serves as a specific biomarker for *in vivo* coagulation activation and subsequent fibrinolysis (Riley et al., 2016; Favresse et al., 2018).

D-dimer has been widely utilized in the diagnosis and therapeutic evaluation of various diseases, particularly as a well-established diagnostic and risk-stratification tool for venous thromboembolism (VTE), which includes deep vein thrombosis (DVT) and pulmonary embolism (PE). Elevated D-dimer levels are strongly associated with an increased short-term risk of VTE (Anderson et al., 2022; Chinese Society on Thrombosis and Hemostasis, 2023) and can also be applied to assess the risk of VTE recurrence and guide the duration of anticoagulant therapy (Franchini et al., 2024). Furthermore, numerous clinical studies have validated D-dimer as a prognostic parameter across various diseases. In the field of infectious diseases, research by Li et al. demonstrated that higher D-dimer levels were independently associated with an increased 28-day mortality risk in elderly sepsis patients (Li et al., 2022) (OR = 1.909, 95% CI: 1.367–2.666, P < 0.001). Other studies have similarly indicated a link between elevated D-dimer and poor prognosis in sepsis (Han et al., 2021; Zhao et al., 2023; Zhang et al., 2024). A meta-analysis revealed that in COVID-19 patients, the pooled sensitivity of D-dimer (Zhan et al., 2021) for predicting disease severity and mortality was 77% (95% CI: 73%–80%) and 75% (95% CI: 65%–82%), respectively, with corresponding specificities of 71% (95% CI: 64%–77%) and 83% (95% CI: 77%–87%). Among oncology patients, multiple studies have shown a positive correlation between elevated D-dimer and mortality in conditions such as lung cancer (Ma et al., 2021) and acute lymphoblastic leukemia (Anderson et al., 2022). Additionally, D-dimer levels have been significantly associated with mortality in patients with spontaneous intracerebral hemorrhage (Shang and Li, 2024) and cardio-cerebrovascular diseases (Deng et al., 2016; Nickel et al., 2021). Against this background, the present study is the first to establish the independent prognostic value of elevated D-dimer specifically in patients with KP-BSI.

The biological mechanisms linking elevated D-dimer to adverse outcomes in KP-BSI patients are not fully understood. This association is thought to arise from a complex interplay between coagulation-fibrinolysis activation, systemic inflammation, and microbial virulence factors. First, endotoxins such as LPS released by *K. pneumoniae* can activate monocytes via Toll-like receptors, promoting the release of pro-inflammatory cytokines (e.g., IL-6, TNF-α) and inducing tissue factor (TF) expression, thereby initiating the extrinsic coagulation pathway (Franchini et al., 2024; Hu et al., 2025). Concurrently, inflammatory mediators can suppress the fibrinolytic system (e.g., by upregulating plasminogen activator inhibitor-1, PAI-1), leading to the accumulation of fibrin degradation products, including D-dimer (Chinese Society on Thrombosis and Hemostasis, 2023; Abbas et al., 2024; Tayal et al., 2024). Second, in the setting of infection, extensive crosstalk exists between the coagulation and inflammatory pathways. Within the inflammatory microenvironment, soluble fibrin oligomers can form microthrombi in the microvasculature, which further activates plasminogen (via tissue-type plasminogen activator, t-PA) to plasmin, degrading cross-linked fibrin and generating D-dimer (Udovenko et al., 2023). Endothelial injury exposes subendothelial collagen, activating platelets and coagulation factor XII and exacerbating thrombus formation (Tayal et al., 2024; Zhao et al., 2024). Moreover, D-dimer itself may act as an inflammatory modulator by upregulating chemokines (e.g., CXCL5, CXCL6) in monocytes and T cells, activating the IL-17 and PI3K-Akt signaling pathways, and thereby promoting immune cell recruitment and amplification of the inflammatory cascade (Hu et al., 2025). Additionally, the capsular polysaccharide of *K. pneumoniae* can directly activate the complement system and coagulation factor XII, accelerating thrombin generation and further promoting a hypercoagulable state (Chinese Society on Thrombosis and Hemostasis, 2023; Yen et al., 2023).

Several limitations of this single-center retrospective analysis must be acknowledged. First, although known confounders were adjusted for using multivariable regression, residual confounding may persist. Second, as a single-center study conducted in a tertiary referral center, our cohort was enriched for severe disease (ICU admission, 63.5%; CRKP, 31.1%), resulting in a high in-hospital mortality rate (47.7%). This referral bias limits the generalizability of our findings to milder or community-onset KP-BSI cases. Third, all variables, including D-dimer, were measured within 24 hours after blood culture collection and do not reflect their dynamic changes during hospitalization, which could affect the accuracy of the findings. Fourth, this study demonstrates an association between D-dimer levels and patient outcomes but cannot establish causality. Finally, our primary outcome was in-hospital mortality rather than 28-day or 30-day mortality, which may limit direct comparability with studies using fixed time-point endpoints. Despite this limitation, we employed multiple analytical strategies, including logistic regression and subgroup analyses, to verify the robustness of this association. Future research is warranted to further elucidate the mechanistic role of D-dimer in the clinical outcomes of patients with KP-BSI. Future multicenter prospective studies will be pivotal in validating the utility of this biomarker, which is routinely accessible, for the early detection of high-risk individuals and to refine subsequent treatment approaches.

## Conclusion

In summary, the D-dimer level measured at the time of blood culture collection is significantly associated with the prognosis of KP-BSI patients. Specifically, elevated levels correlate with higher in-hospital mortality. Consequently, D-dimer serves as a readily accessible biomarker that can identify high-risk individuals and functions as an independent predictor of clinical outcomes.

## Data Availability

The raw data supporting the conclusions of this article will be made available by the authors, without undue reservation.

## Supplementary material

Supplementary Figure 1 Receiver operating characteristic (ROC) curve of D-dimer for predicting in-hospital mortality in patients with Klebsiella pneumoniae bloodstream infection.

Supplementary Table 1 Sensitivity analysis using the Youden index-derived D-dimer cutoff (0.46 mg/L).
