# Supplementary figures and images for: Elevated D-dimer is independently associated with in-hospital mortality in patients with Klebsiella pneumoniae bloodstream infection: a cohort study
Source: Front Cell Infect Microbiol. 2026 Jul 6;16:1778926. doi: 10.3389/fcimb.2026.1778926 (PMC13381231; doi:10.3389/fcimb.2026.1778926)

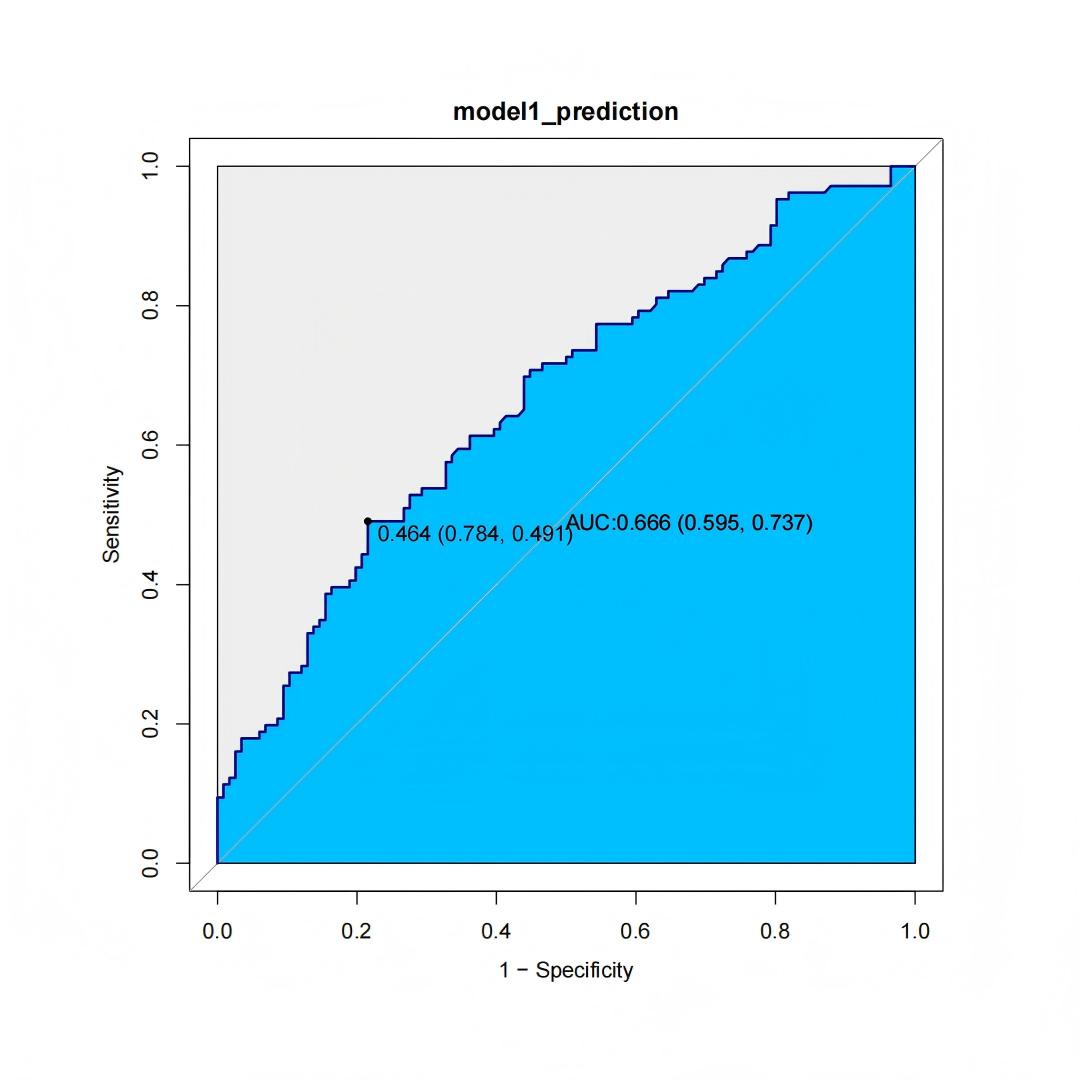

Supplement: Supplementary Figure 1 — Receiver operating characteristic (ROC) curve of D-dimer for predicting in-hospital mortality in patients with Klebsiella pneumoniae bloodstream infection. [file Image1.jpg]
