# Supplementary material for: Elevated D-dimer is independently associated with in-hospital mortality in patients with Klebsiella pneumoniae bloodstream infection: a cohort study
Source: Front Cell Infect Microbiol. 2026 Jul 6;16:1778926. doi: 10.3389/fcimb.2026.1778926 (PMC13381231; doi:10.3389/fcimb.2026.1778926)
Supplement: Supplementary Table 1 — Sensitivity analysis using the Youden index-derived D-dimer cutoff (0.46 mg/L). [file Table1.docx]

Table S1. Sensitivity analysis using the Youden index-derived D-dimer cutoff (0.46 mg/L).

| **Variable** | **N.total** | **n.event_%** | **Crude Model** | | **Adjusted Model** | |
| --- | --- | --- | --- | --- | --- | --- |
|  |  |  | HR (95% CI) | p | HR (95% CI) | p |
| **D-dimer <0.46(mg/L)** | 27 | 5 (18.5) | 1(Ref) |  | 1(Ref) |  |
| **D-dimer ≥0.46(mg/L)** | 195 | 101(51.8) | 3.02 (1.22–7.45) | 0.017 | 0.76 (0.27–2.11) | 0.596 |

Adjusted for age, sex, WBC, CRP, Alb, APTT, diabetes, malignancy, chronic liver disease, chronic renal disease, SOFA, ICU admission, inappropriate initial therapy, and CRKP.
